# Sudden pediatric death unveiling pulmonary arteriovenous malformations

**DOI:** 10.4322/acr.2024.489

**Published:** 2024-05-22

**Authors:** Hadeel Abu-El-Rub, Rashed Shatnawi, Yahia I AbuZetun, Doaa Ghorab, Ali M. Shotar

**Affiliations:** 1 Yarmouk University, Forensic Medicine and Toxicology Unit, Department of Clinical Sciences, Faculty of Medicine, Irbid, Jordan; 2 Mansoura University, Pathology Department, Faculty of Medicine, Mansoura, Egypt; 3 Jordan University of Science and Technology, Department of Legal Medicine, Toxicology and Forensic Science, School of Medicine, Irbid, Jordan

**Keywords:** Arteriovenous Malformations, Sudden Death, Forensic Pathology, Autopsy

## Abstract

Pulmonary arteriovenous malformations (PAVMs) are abnormal vascular connections between pulmonary arteries and veins, often associated with hereditary hemorrhagic telangiectasia (HHT). Most PAVMs are asymptomatic, but life-threatening complications like pulmonary hemorrhage, brain abscesses, and paradoxical emboli can emerge, so prompt diagnosis and treatment are crucial. We report a case of sudden pediatric death in a two-year-old female with no past medical history. Initial vomiting and fast deterioration resulted in a sudden cardiac arrest. The postmortem examination found histological evidence of consistent, extensive lung damage. The absence of the characteristic symptoms made for some challenges when it came to diagnosis, showing precisely that in early life, you could well have many difficulties in catching PAVMs. This case highlights the need to take PAVMs into account as a potential cause of sudden death, particularly when there are no conspicuous symptoms. Awareness among forensic pathologists and consideration of genetic analysis for HHT in such cases is crucial for accurate diagnosis and management.

## INTRODUCTION

Pulmonary arteriovenous malformations (PAVMs) are anomalous vascular connections directly linking pulmonary arteries and veins, circumventing capillary beds, and forming continuous intrapulmonary right-to-left shunts characterized by low resistance and high flow.^1-3^ Most PAVM cases (80%–90%) are linked to hereditary hemorrhagic telangiectasia (HHT), with a smaller subset representing sporadic occurrences.^4^ While a significant number of PAVM patients may be asymptomatic, those undiagnosed are at risk of developing severe complications, including ischemic stroke, myocardial infarctions, cerebral abscesses, massive hemoptysis, and hemothorax.^5,6^ PAVMs exhibit growth throughout the individual's life.^7^ The prevalence of PAVMs is approximately twice as high in women as in men, although there is a male predominance among newborns.^8^ The accelerated development of PAVMs can lead to a rapid decline in physical capacity, particularly during pregnancy or shortly after childbirth, due to increased shunting.^9^ This case report highlights the occurrence of a sudden pediatric death attributed to pulmonary arteriovenous malformations (PAVMs).

## CASE REPORT

A two-year-old female without past medical history presented to the ER with a same-day history of severe vomiting. She wasn't dehydrated, and her vital signs were stable, so she was compensated with IV fluids and discharged with instructions to return in case of any complications. Later on the same day, she was readmitted as clinically unstable, with a heart rate of more than 100 beats per minute (BPM) and an oxygen saturation of around 92% on an oxygen mask. Her condition worsened rapidly, leading to a sudden cardiac arrest. Cardiopulmonary resuscitation was performed twice, but unfortunately, there was no response, and she was declared deceased. The body was referred for postmortem examination, and a complete autopsy was performed.

## AUTOPSY PRESENTATION

On external examination, her weight was 13 kg (75th percentile), height 88 cm (81st percentile), and body mass index 16.8 kg/m2. No visible injuries were observed externally. No bleeding was seen on the conjunctiva and sclera, and the mouth was clear from bruises or injuries. The autopsy revealed no scalp injuries or hemorrhage, and no fractures in the skull were identified. The brain weighed 1100 g (normal range: 935–1425) and was edematous, with pronounced congestion of the cerebral vessels, but no lesions or injuries were noted, and there was no evidence of thromboembolism. 100 ml of serous fluids were found in the pleural cavity, but no bleeding or adhesions were seen. The heart weighed 70 g (normal range: 42–96), with healthy coronary arteries and no cardiac malformation. The right and left lungs were severely congested and edematous, weighing 130 g (normal range: 26–262) and 110 g(normal range: 40–208), respectively. No bleeding or fluids were found in the abdomen, and the gastric lining appeared normal, but the stomach contained dark-colored fluids sent for analysis. The liver and spleen were normal, weighing 370 g (normal range: 289–681) and 60 g (normal range: 16–86), respectively. The kidneys weighed 50 g (normal range: 20–70) each had no remarkable findings. All organs were congested, and no other macroscopical abnormalities were observed in the rest of the autopsy.

Toxicological analysis using gas chromatography-mass spectrometry (GC-MS) revealed no drugs, alcohol, or pesticides in the samples taken from the blood, bile, and stomach content. No microbiological, genetic studies or testing for inborn errors of metabolism were done.

**Figure 1:**
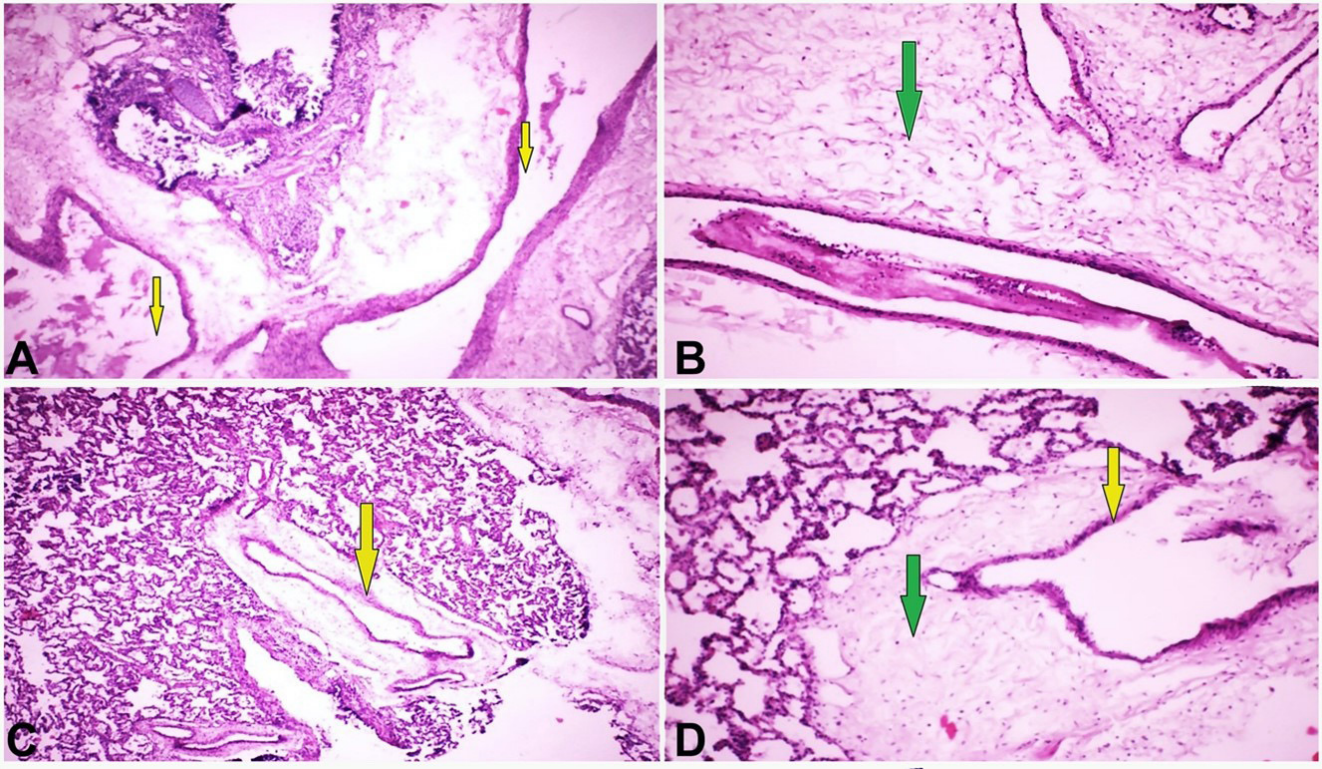
Photomicrographs of the lung. **A**, **B**, **C**, and **D** have different views of arteriovenous malformation involving the lungs; irregularly dilated blood vessels with different thin and thick walls (veins, arteries) labeled with yellow arrows, and the wall of blood vessels shows myxoid degeneration pointed with green arrows (All H and E, X40).

**Figure 2:**
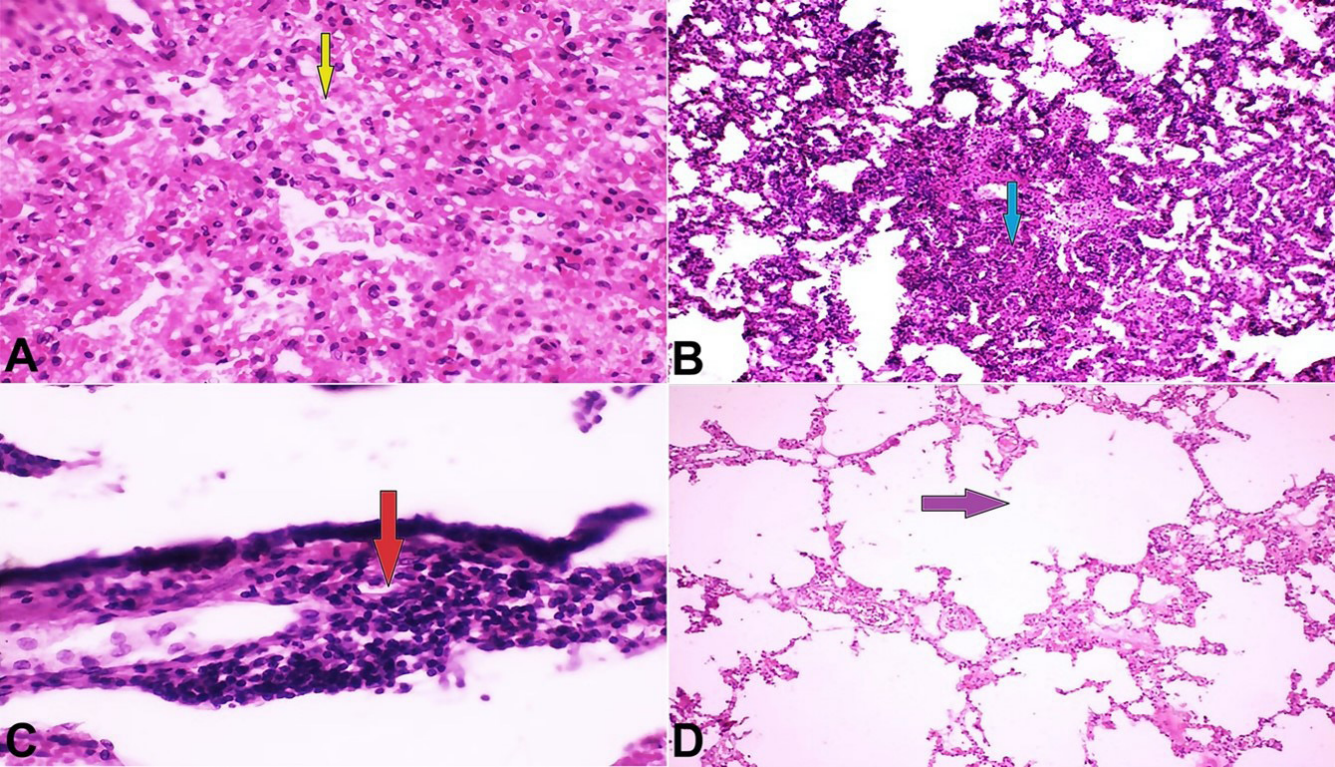
Photomicrographs of Arteriovenous malformations complications in the lung. **A** **–** New capillaries with interstitial hemorrhage pointed with yellow arrow (H& E, X400); **B** **–** interstitial pneumonia; mixed inflammatory cells pointed with blue arrow (H&E, X100); **C** **–** Acute bronchiolitis labeled with red arrow (H& E, X400); **D** **–** Secondary emphysema; dilated lung alveoli with rupture of alveolar septa pointed with purple arrow (H&E, X100).

The pulmonary histological examination showed irregularly dilated blood vessels with thin and thick walls involving veins and arteries. The blood vessel walls showed myxoid degeneration, and new capillary formation was observed with interstitial hemorrhage. Adjacent lung tissue shows secondary interstitial pneumonia consisting of infiltration of alveolar septa with mixed inflammatory cells (neutrophils and lymphocytes), acute bronchiolitis formed of infiltration of the wall of bronchioles with acute inflammatory cells, and secondary emphysema was seen as dilated lung alveoli with ruptured alveolar septa in between (Figures 1 and 2).

**Figure 3:**
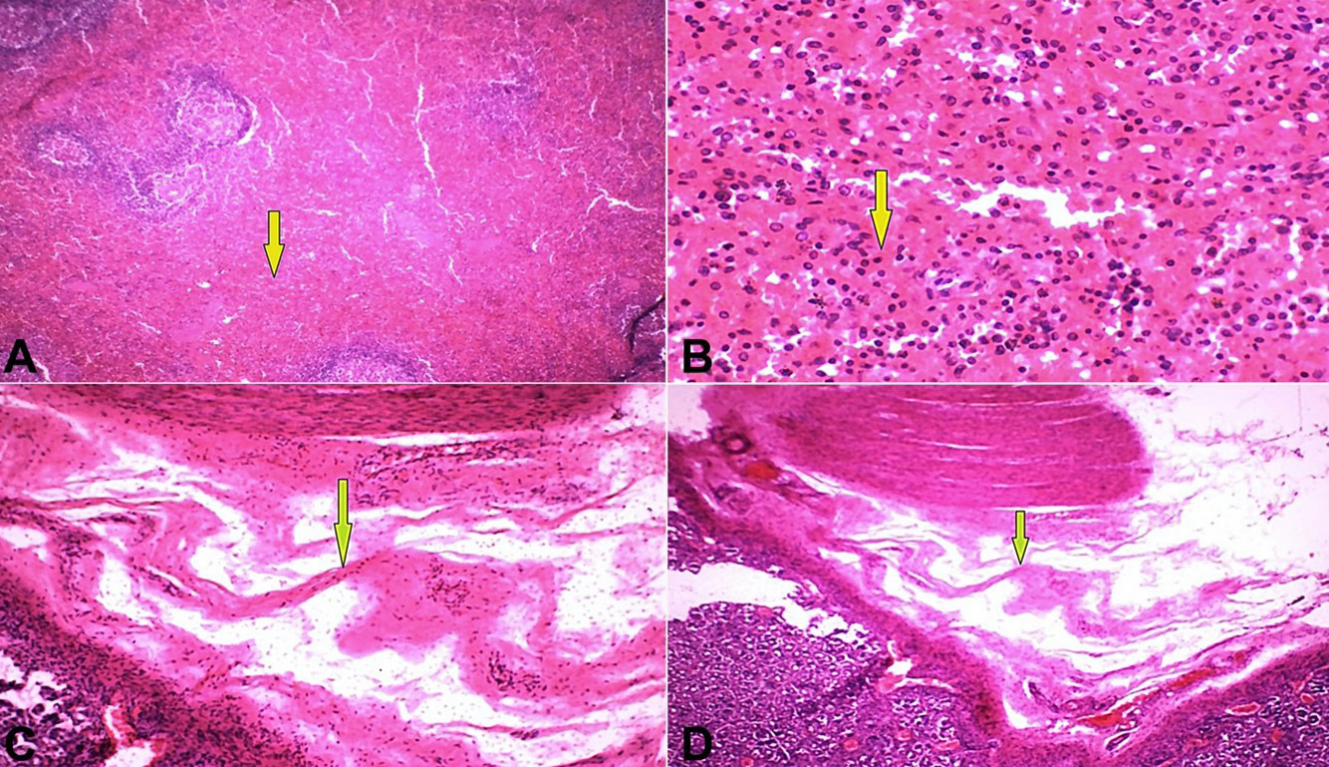
Photomicrographs of: **A** **–** Spleen - Congestion; the red pulp is widened with atrophy of white pulp labeled with yellow arrow (H& E, X40); **B –** Spleen - High power of congested red pulp pointed with yellow arrow (H& E, X400); **C –** Stomach -Arteriovenous malformation involving muscle wall; irregularly dilated blood vessels with different wall thickness splitting the muscle wall and showing hemorrhage pointed with green arrow (H& E, X100); **D –** Stomach - High power of arteriovenous malformation involving the wall pointed with green arrow (H&E, X400).

The spleen exhibited congestion with hemorrhage, with widened and congested red pulp and atrophy of white pulp (Figures 3A and 3B). The stomach sections also showed arteriovenous malformation seen as irregularly dilated blood vessels with different wall thicknesses, splitting the muscle wall, and hemorrhage (Figures 3C and 3D).

Death was attributed to the pulmonary hemorrhage due to PAVMs.

## DISCUSSION

Diagnosing cases in the emergency room is challenging without signs and symptoms that potentially lead to hazardous outcomes; moreover, if patients fail to recognize these symptoms as indicative of a pathological entity. The absence of self-identified signs, particularly in congenital conditions like arteriovenous malformations (AVMs), may escalate into a life-threatening situation. Pulmonary arteriovenous malformations, noted in numerous textbooks as a potential cause of hemoptysis, posed diagnostic challenges, and a delayed diagnosis occurred in our case.^10^ The nature of hemoptysis complicates the diagnosis of pulmonary arteriovenous malformation based solely on this symptom. Patients exhibiting symptoms like typical chest x-ray findings, mucocutaneous telangiectasias, unexplained exertional dyspnea, cyanosis, clubbing (in severe cases), or hemoptysis should raise suspicion of pulmonary arteriovenous malformations.^10^ Due to the substantial risk of complications exceeding 50% and higher during pregnancy, prompt treatment of pulmonary arteriovenous malformations is imperative to preclude complications.^11^ In our case, the absence of symptoms other than vomiting precluded the diagnosis until post-mortem examination, which revealed severe and consistent lung involvement. Macroscopic pulmonary AVMs on chest radiographs typically manifest as spherical, lobulated masses, primarily in the lower lobes. The observed gross lung features align with findings in other studies, including increased lung weight, congestion, and edema. The initial presentation of pulmonary AVM might involve extensive hemoptysis or hemothorax, with a definitive diagnosis confirmed through histopathological examination after surgical intervention. Isolated acute vomiting is insufficient for diagnosis, making the identification of PAVM quite impossible. The frequent misdiagnosis of PAVM in early life highlights the importance of increasing autopsy numbers for epidemiological contributions.^12,13^ HHT is a widely recognized hereditary autosomal dominant inherited disorder characterized by diverse multisystemic abnormalities in vascular tissue. The Curaçao criteria, which aid in the diagnosis, include (i) spontaneous and recurrent epistaxis, (ii) multiple telangiectasias on the skin and mucous membranes in specific locations (such as the tongue, mouth/throat, lips, conjunctivae, ears, hands/fingers, and gastrointestinal tract), (iii) the presence of visceral arteriovenous malformations (AVMs) in organs such as the lung, liver, brain, and spine, and (iv) a positive family history (having a first-degree family member with HHT).^14^ In our case, aside from the arteriovenous malformations in the lungs and the stomach, none of the diagnostic criteria for HHT were observed. Consequently, genetic testing was not conducted.

In conclusion, this case underscores pulmonary arteriovenous malformations as potential contributors to sudden, unexpected fatalities, emphasizing the need for pathologists to be aware of such a cause and to appreciate the need for genetic analysis when suspecting HHT.

## References

[1] Shovlin CL, Condliffe R, Donaldson JW, Kiely DG, Wort SJ, British Thoracic Society (2017). British Thoracic Society clinical statement on pulmonary arteriovenous malformations. Thorax.

[2] Saboo SS, Chamarthy M, Bhalla S (2018). Pulmonary arteriovenous malformations: diagnosis. Cardiovasc Diagn Ther.

[3] Contegiacomo A, del Ciello A, Rella R (2019). Pulmonary arteriovenous malformations: what the interventional radiologist needs to know. Radiol Med (Torino).

[4] Lee EY, Boiselle PM, Cleveland RH (2008). Multidetector CT evaluation of congenital lung anomalies. Radiology.

[5] Gossage JR, Kanj G (1998). Pulmonary arteriovenous malformations. A state of the art review. Am J Respir Crit Care Med.

[6] Pollak JS, Saluja S, Thabet A, Henderson KJ, Denbow N, White RI (2006). Clinical and anatomic outcomes after embolotherapy of pulmonary arteriovenous malformations. J Vasc Interv Radiol.

[7] Vase P, Holm M, Arendrup H (1985). Pulmonary arteriovenous fistulas in hereditary hemorrhagic telangiectasia. Acta Med Scand.

[8] Allen SW, Whitfield JM, Clarke DR, Sujansky E, Wiggins JW (1993). Pulmonary arteriovenous malformation in the newborn: A familial case. Pediatr Cardiol.

[9] Shovlin CL, Winstock AR, Peters AM, Jackson JE, Hughes JM (1995). Medical complications of pregnancy in hereditary haemorrhagic telangiectasia. QJM.

[10] Minamikawa R, Ryu Y, Sanada J, Takata H, Okumura T (2020). Pulmonary arteriovenous malformations diagnosed through hemoptysis: a case report. Radiol Case Rep.

[11] Pierucci P, Murphy J, Henderson KJ, Chyun DA, White RI (2008). New definition and natural history of patients with diffuse pulmonary arteriovenous malformations: twenty-seven-year experience. Chest.

[12] Aggarwal V, Khan DM, Rhodes JF (2017). Pulmonary arteriovenous malformation causing systemic hypoxemia in early infancy. Case Rep Pediatr.

[13] Mohammed MHA, Hrfi A, AlQwee AM, Tamimi O (2018). Pulmonary arteriovenous malformation in a neonate: a condition commonly misdiagnosed. Sudan J Paediatr.

[14] Ishikawa T, Pollak S, Pflugradt R (2010). Pulmonary arteriovenous malformation causing sudden death due to spontaneous hemothorax. Int J Legal Med.

